# Fatal disseminated infection due to *Sarocladium kiliense* in a diabetic patient with COVID‐19

**DOI:** 10.1002/ccr3.4596

**Published:** 2021-09-27

**Authors:** Maryam Ranjbar‐Mobarake, Jamileh Nowroozi, Parisa Badiee, Sayed Nassereddin Mostafavi, Rasoul Mohammadi

**Affiliations:** ^1^ Department of Microbiology North branch Islamic Azad University Tehran Iran; ^2^ Clinical Microbiology Research Center Shiraz University of Medical Sciences Shiraz Iran; ^3^ Infectious Diseases and Tropical Medicine Research Center Isfahan University of Medical Sciences Isfahan Iran; ^4^ Department of Medical Parasitology and Mycology School of Medicine, Infectious Diseases and Tropical Medicine Research Center Isfahan University of Medical Sciences Isfahan Iran

**Keywords:** COVID‐19, diabetic patient, disseminated infection, *Sarocladium kiliense*

## Abstract

*Sarocladium kiliense* is a soil saprophytic mold with worldwide distribution, which can infect humans and other mammals, sporadically. The clinical manifestations include mycetoma, onychomycosis, keratomycosis, pneumonia, and arthritis. Here, we present a disseminated infection due to *S. kiliense* in a diabetic patient infected to coronavirus disease 2019 (COVID‐19) from Isfahan, Iran.

## Graphical abstract

We alarm co‐infection with opportunistic fungi among patients with COVID‐19. It can increase the mortality rate significantly, especially among immunocompromised patients.

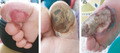

## 1 INTRODUCTION

*Sarocladium kiliense*, formerly known as *Acremonium kiliense*, ^1^ is an omnipresent soil saprophytic fungus generally found in the environment such as cereal fields and the soils of grass lands, and sporadically infecting humans and other mammals.^2^, ^3^, ^4^ The species of *Sarocladium* are morphologically very homologous and in the most of the clinical cases the causative agent is reported only as a *Sarocladium*/*Acremonium* sp., which dramatically decreases the value of the investigations.^5^ This is the principal cause that the actual incidence of the various species of *Sarocladium* in the clinical setting is unknown. Molecular identification of *Sarocladium* using modern DNA‐based techniques is essential for a critical assessment of the reported cases. This fungus can cause opportunistic infections, such as mycetoma, onychomycosis, fungal keratitis, in immunocompetent individuals, and osteomyelitis, pneumonia, arthritis, peritonitis, endocarditis, meningitis, and sepsis in immunocompromised patients.^6^, ^7^ The main risk factors are considered as the use of catheters and prosthesis, anatomic disorders, immunosuppressive therapy, autoimmune diseases, diabetes mellitus, and malignancies.^2^, ^8^ Here, we present a systemic case of *S*. *kiliense* in a diabetic patient infected to coronavirus disease 2019 (COVID‐19) from Isfahan, Iran.

## 2 CASE PRESENTATION

**FIGURE 1:**
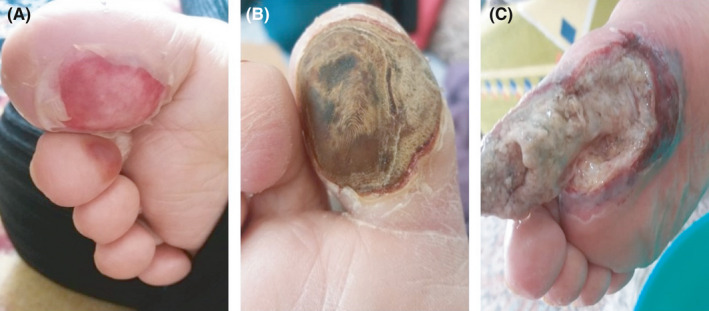
Primary lesion in the big toe A, the lesion after treatment with medicinal herbs B, progressive lesion (grade 3, stage D) for which the patient referred to the hospital C

**FIGURE 2:**
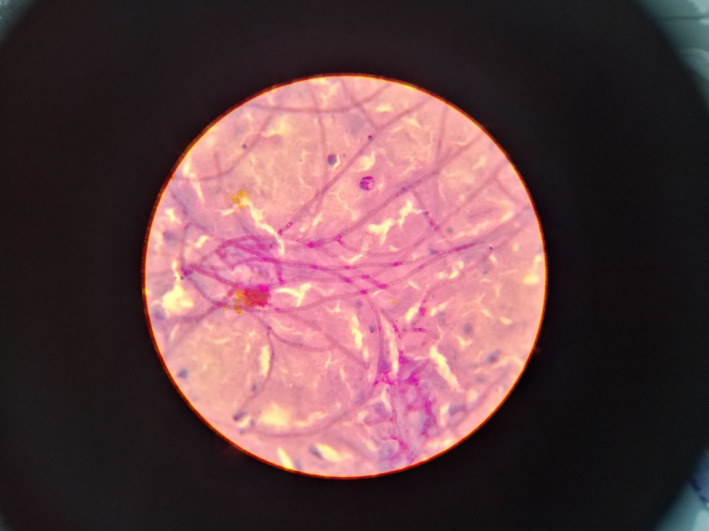
Septate hyaline fungal hyphae in histopathological findings, Periodic Acid‐Schiff (PAS) Stain, original magnification ×40

**FIGURE 3:**
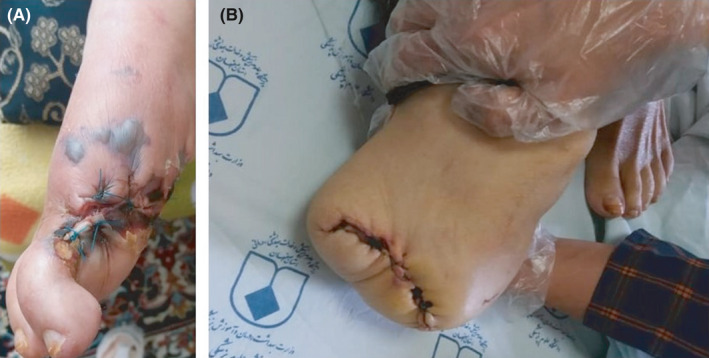
Amputation of big toe due to the grade 3, stage D lesion A, amputation of foot because of progressive necrotic lesion B

**FIGURE 4:**
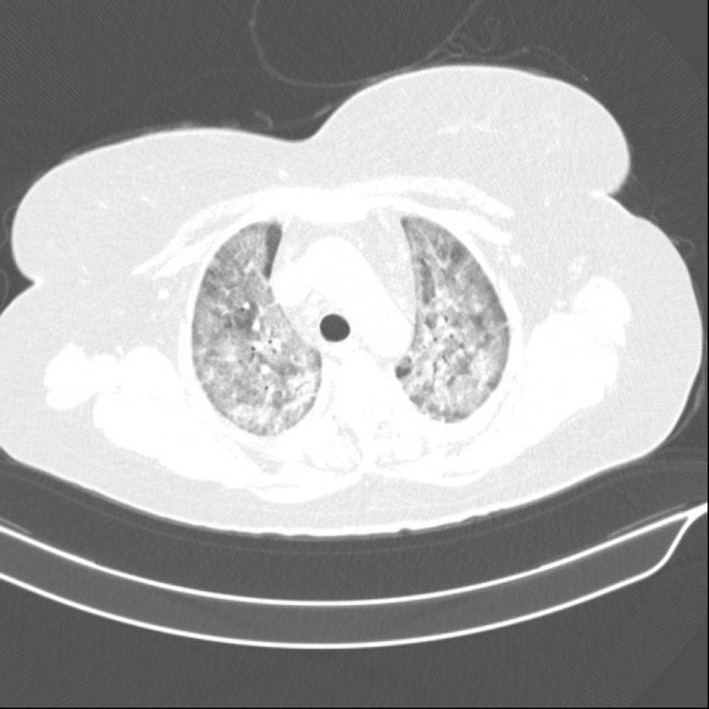
Chest computed tomography (CT) scan revealed diffuse ground‐glass opacity in both lungs with crazy‐paving in favor of COVID‐19 pneumonia

**FIGURE 5:**
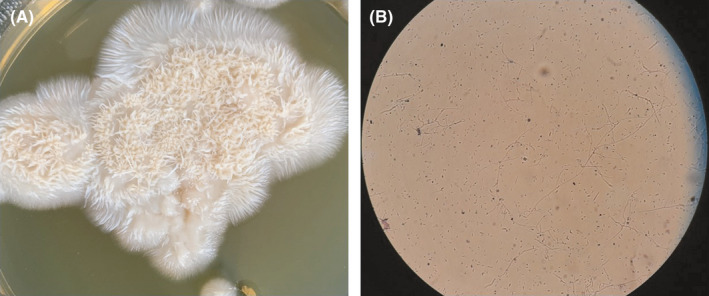
White to pink slow‐growing colonies of *S*. *kiliense* that often compact and moist at first, then becoming floccose or suede‐like with age A, fine and hyaline hyphae producing simple awl‐shaped erect phialides with one‐celled hyaline conidia B

A 74‐year‐old woman with 25 years history of diabetes mellitus was referred to a private traditional medicine center due to a lesion on her toe (Figure 1A). Because of diabetes, she went blind when she was 59 years old. She used medicinal plants for 14 days to treat her wound; however, it got worse (Figure 1B). On August 7, 2020, she was referred to the Gharazi Hospital, Isfahan, Iran, with fever (38°C) and a progressive lesion (grade 3, stage D; Figure 1C). She was admitted to the Internal Medicine Department. Her medical checkup findings were as follows: respiratory rate (RR): 32 breaths per minute, heart rate (HR): 120 beats per minute, blood pressure (BP): 120/80 mmHg, and oxygen saturation (SpO2): 95% in room air. She was relatively conscious; however, she suffered from shortness of breath. Hematological and biochemical tests were summarized in Table 1. Regular insulin was started to decrease blood sugar, and Targocid (6 mg/kg/12 h) with Tazocin (4.5 g/8 h) was also applied for her. On August 9, 2020, septate hyaline fungal hyphae were observed in histopathological findings (Figure 2). At this stage, aspergillosis and fusariosis were differential diagnosis. On August 10, 2020, her toe was amputated (Figure 3A) and liposomal amphotericin B (AmBisome; 5 mg/kg/day) was added to her regimen. Six days later, because of necrosis, her foot was amputated from the upper part (Figure 3B). Due to the dyspnea and oxygen saturation of 80% in room air, chest computed tomography (CT) scan was done and demonstrated COVID‐19 pneumonia (Figure 4). Real‐time reverse transcriptase‐polymerase chain reaction (rRT‐PCR) confirmed SARS‐CoV‐2 infection. Oxygen therapy with nasal cannula (4 L/min), methylprednisolone 130 mg for 4 days, and treatment with interferon Beta‐1b 0.25 mg SQ every 48 h for four dosages were started for her. On August 17, 2020, she transferred to the intensive care units (ICUs) of Chamran University Hospital, Isfahan, Iran, for better management of COVID‐19 infection. She had fever (39.5°C), and one blood sample was taken for probable systemic infection. Due to the severe dyspnea and SpO2 60%, she was intubated on August 19, 2020; however, she died the same day. Four days after death, *Sarocladium* spp. recovered from blood culture (Figure 5) and PCR‐sequencing was applied for identification. ITS1‑5.8SrDNA‑ITS2 region was amplified using ITS1 (5′‑TCC GTA GGT GAA CCT GCG G‑3′) and ITS4 (5′‑TCC TCC GCT TAT TGA TAT GC‑3′) primers and was subjected to sequence analysis in a forward direction (Bioneer). The sequence product was analyzed with Chromas 2.4 (https://chromas.software.informer.com/2.4/) and then evaluated using the NCBI BLAST searches against fungal sequences existing in DNA databases (https://blast.ncbi.nlm.nih.gov/Blast.cgi). The ITS gene sequence was deposited in the GenBank under the accession number MW679681. This research was approved by the Ethics Committee of Isfahan University of Medical Science (no. IR.MUI.MED.REC.1399.912), and written informed consent was obtained from the patient.

**TABLE 1:** Hematological and biochemical tests of the current patient after admission to the hospital

Hematology		
Test	Result	Unit
WBC	8.9	×10^3^/µl
RBC	3.98	×10^3^/µl
Hemoglobin	10.6	g/dl
HCT	34.2	%
MCV	85.9	fl
MCH	26.6	pg
MCHC	31	g/dl
Neutrophils	7.6	×10^3^/ml
Platelet	288	×10^3^/ml
ESR	70	mm/h

Biochemistry		
Test	Result	Unit
FBS	350	mg/dl
Creatinine	1.1	mg/dl
Sodium	147	mmol/L
Potassium	3.5	mmol/L
Magnesium	1.7	mg/dl

## 3 DISCUSSION

The emergence of uncommon human and animal opportunistic fungi, such as *Sarocladium*, definitely impresses severely immunosuppressed patients and needs a high level of clinical attention. *Sarocladium* genus contains several of morphologically and genetically mold fungi that are commonly found in the environment. The most of species of this genus are opportunistic pathogens of plants and soil saprobes.^5^ Within the genus, *S*. *kiliense* is the most prevalent pathogen in human clinical reports, producing predominantly mycetomas^9^; however, other critical cases affecting lungs, nails, joints, or catheter‐related bloodstream infections are available.^10^, ^11^ The most fungemia related to *S*. *kiliense* have been reported from Colombia and Chile.^12^ Fever, maculopapular rash, tachycardia, and hematuria are popular symptoms of bloodstream infections in a great number of patients,^13^, ^14^ but our patient only had fever. Hematological malignancies, solid organ transplants, solid tumors, renal transplantation, and Crohn's disease are main predisposing factors for disseminated infection with *Sarocladium* spp.^14^, ^15^, ^16^ ^;^ nevertheless, the present case was diabetic with no abovementioned risk factors. Blood culture is essential for diagnosis of *Sarocladium* blood infections, because in almost all studies, ^7^, ^14^, ^15^, ^17^ the fungus has been isolated from blood culture, similar to the present case. Bloodstream infections due to *Sarocladium* are usually treated with various medications such as amphotericin B (AMB) and voriconazole.^15^, ^18^ Many patients recover after changing regimen to voriconazole following AMB failure. Unfortunately, voriconazole did not use for the present patient, and she died 9 days after taking AmBisome. In this connection, we highlight the need for antifungal susceptibility testing (AFST) of clinical isolates caused fungemia for selecting the best treatment, since empirical therapy with AMB failed in great number of patients.^14^ One of the major limitations of the present investigation was the lack of AFST for current strain. Timely treatment and removal of catheter as the source of infection, if possible, were also substantial steps to overcome the infection. *Sarocladium* cutaneous infections have been reported in the literature^19^, ^20^, ^21^; however, the fungus has been molecularly identified only in one case (*S*.* strictum*).^19^ Reports of *Sarocladium* superficial and subcutaneous infections were recorded from India,^19^ Turkey,^20^ Russia,^22^ Taiwan,^23^ France,^24^ Brazil,^25^ and Korea.^26^ To our knowledge, this is the first report of systemic *Sarocladium* infection from Iran that has disseminated to the skin. The clinical features of cutaneous infections include painless swelling, pustules and nodules, scaly plaque, redness, ulceration, necrotic areas, and purulent exudate.^23^, ^27^ The cutaneous lesion of the current case was necrotic and revealed a lot of purulent exudate with no pustules, nodules, or scaly plaques. Diagnosis is based predominantly on skin biopsy, which presents a typical granulomatous reaction with giant cells in histopathological examination, and the presence of hyaline fungal hyphae.^16^ Since the skin sample was taken from necrotic lesions; so, we could not see activated macrophages and giant cells in histopathological reaction (Figure 2). Similar to the current case, Khan et al^28^ presented a case of *Sarocladium* infection in a patient with long history of diabetes. They isolated *S*. *kiliense* from the peritoneal fluid, and E‐test was performed to determine drug susceptibility. The isolate was susceptible to posaconazole and voriconazole but resistant to caspofungin and amphotericin B. Although, voriconazole therapy was successful; however, he died due to severe bacterial sepsis. Fernández‐Silva et al^8^ suggested combination therapy for *S*. *kiliense* infections. They revealed that voriconazole + anidulafungin and posaconazole + anidulafungin had the most synergism and antagonism effects, respectively. Despite amphotericin B can be effective for lesions of the skin and soft tissues; nevertheless, the use of this antifungal alone in cases of disseminated infections is controversial.^29^ In immunocompromised patients, prognosis of infection depends on the underlying disease, duration of disease, and clinical form of infection. In this regard, survival rate in patients with local, invasive, and disseminated forms is 100%, 75%, and 50%, respectively. ^29^, ^30^ Corticosteroid therapy, lymphopenia, and dysregulation of immune responses are known main risk factors for fungal infection among COVID‐19 patients.^31^ In the present case, methylprednisolone (130 mg) was applied for 4 days which can be an important risk factor for fungal infections. Riche et al^32^ showed high frequency of *Candida* bloodstream infection among COVID‐19 patients receiving corticosteroids in Brazil. Despite antifungal therapy, mortality rate was 72.7% in their survey. The frequency of fungal co‐infections in COVID‐19 patients has still been rarely studied, and more investigations should be performed among vulnerable population such as ICU patients, diabetic patients, and patients with hematological malignancies. Aspergillosis,^33^ mucormycosis,^31^ candidiasis,^32^ histoplasposis,^34^ and cryptococcosis^35^ are reported fungal infections among COVID‐19 patients; however, to our knowledge, this is the first case of *Sarocladium* infection in a COVID‐19 patient.

## 4 CONCLUSION

We highlight the early diagnosis, accurate fungal identification, precise and adequate treatment of hyalohyphomycosis to avoid serious effects of this infection especially in COVID‐19 patients who are taking corticosteroids. Since empirical therapy with amphotericin B failed in the most patients, antifungal susceptibility testing of the clinical isolates is strongly recommended for better management of this fatal infection.

## CONFLICT OF INTEREST

None declared.

## AUTHOR CONTRIBUTIONS

MRM and SNM contributed to data acquisition and providing the clinical figures. MRM, JN, PB, and RM contributed to identifying *Sarocladium kiliense* using phenotypic and molecular methods, and providing the mycological illustrations. RM served as the corresponding author and designed and supervised all the aspects and contributed to manuscript editing.

## CONSENT STATEMENT

Published with written consent of the patient.

## Data Availability

The ITS gene sequence of Sarocladium kiliense was deposited in the GenBank under the accession number MW679681.

## References

[1] Summerbell R, Gueidan C, Schroers H, et al. Acremonium phylogenetic overview and revision of gliomastix, sarocladium, and trichothecium. Stud Mycol. 2011;68:139‐162. 21523192 10.3114/sim.2011.68.06 PMC3065988

[2] Pastorino AC, Menezes UPD, Marques HHdS, et al. Acremonium kiliense infection in a child with chronic granulomatous disease. Braz J Infect Dis. 2005;9(6):529‐534. 16410951 10.1590/s1413-86702005000600014

[3] Klimko N, Khostelidi S, Melekhina Y, Gornostaev D, Semelev V. Acremonium pneumonia successfully treated in patient with acute myeloid leukemia: a case report. J Bacteriol Mycol. 2016;2(5):116‐119.

[4] Fernández‐Silva F, Capilla J, Mayayo E, Sutton D, Guarro J. Experimental murine acremoniosis: an emerging opportunistic human infection. Med Mycol. 2014;52(1):29‐35. 24577339 10.3109/13693786.2013.797610

[5] Perdomo H, Sutton DA, García D, et al. Spectrum of clinically relevant acremonium species in the United States. J Clin Microbiol. 2011;49(1):243‐256. 21068274 10.1128/JCM.00793-10 PMC3020405

[6] Lopes JO, Rolling LC, Neumaier W. Kerionlike lesion of the scalp due to acremonium kiliense in a noncompromised boy. Rev Inst Med Trop. 1995;37(4):365‐368. 10.1590/s0036-46651995000400015 8599069

[7] Mattei D, Mordini N, Nigro L, et al. Successful treatment of acremonium fungemia with voriconazole. Mycoses. 2003;46(11–12):511‐514. 14641626 10.1046/j.0933-7407.2003.00924.x

[8] Fernández‐Silva F, Capilla J, Mayayo E, Sutton D, Guarro J. In vitro evaluation of antifungal drug combinations against sarocladium (Acremonium) kiliense, an opportunistic emergent fungus resistant to antifungal therapies. Antimicrob Agents Chemother. 2014;58(2):1259‐1260. 24247142 10.1128/AAC.02131-13 PMC3910885

[9] Venugopal PV, Venugopal TV. Pale grain eumycetomas in Madras. Australas J Dermatol. 1995;36(3):149‐151. 7487741 10.1111/j.1440-0960.1995.tb00957.x

[10] Júnior MC, de Moraes AA, Silva HM, Costa CR, Silva MdRR. Acremonium kiliense: case report and review of published studies. Mycopathologia. 2013;176(5):417‐421. 24002104 10.1007/s11046-013-9700-x

[11] Ioakimidou A, Vyzantiadis T‐A, Sakellari I, et al. An unusual cluster of acremonium kiliense fungaemias in a haematopoietic cell transplantation unit. Diagn Microbiol Infect Dis. 2013;75(3):313‐316. 23290506 10.1016/j.diagmicrobio.2012.11.015

[12] Etienne KA, Roe CC, Smith RM, et al. Whole‐genome sequencing to determine origin of multinational outbreak of sarocladium kiliense bloodstream infections. Emerg Infect Dis. 2016;22(3):476‐481. 26891230 10.3201/eid2203.151193 PMC4766898

[13] Warris A, Wesenberg F, Gaustad P, Verweij PE, Abrahamsen TG. Acremonium strictum fungaemia in a paediatric patient with acute leukaemia. Scand J Infect Dis. 2000;32(4):442‐444. 10959664 10.1080/003655400750045132

[14] Hitoto H, Pihet M, Weil B, Chabasse D, Bouchara J‐P, Rachieru‐Sourisseau P. Acremonium strictum fungaemia in a paediatric immunocompromised patient: diagnosis and treatment difficulties. Mycopathologia. 2010;170(3):161‐164. 20340045 10.1007/s11046-010-9306-5

[15] Rodríguez ZC, Ramos MG. Acremonium species associated fungemia: a novel pathogen in the immunosuppressed patient. Bol Asoc Med P R. 2014;106(3):29‐31. 25470906

[16] Pérez‐Cantero A, Guarro J. Sarocladium and acremonium infections: new faces of an old opportunistic fungus. Mycoses. 2020;63(11):1203‐1214. 33090564 10.1111/myc.13169

[17] Foell J, Fischer M, Seibold M, et al. Lethal double infection with acremonium strictum and aspergillus fumigatus during induction chemotherapy in a child with ALL. Pediatr Blood Cancer. 2007;49(6):858‐861. 16429409 10.1002/pbc.20756

[18] Díaz‐Couselo FA, Zylberman M. Catheter‐related acremonium kiliense fungemia in a patient with ulcerative colitis under treatment with infliximab. Case Rep Infect Dis. 2011;2011:710740. 10.1155/2011/710740 22567476 PMC3336241

[19] Sharma A, Hazarika N, Barua P, Shivaprakash M, Chakrabarti A. Acremonium strictum: report of a rare emerging agent of cutaneous hyalohyphomycosis with review of literatures. Mycopathologia. 2013;176(5):435‐441. 10.1007/s11046-013-9709-1 24121988

[20] Hilmioglu S, Metin DY, Tasbakan M, Pullukcu H, Akalin T, Tumbay E. Skin infection on both legs caused by acremonium strictum (case report). Ann Saudi Med. 2015;35(5):406‐408. 26506977 10.5144/0256-4947.2015.406 PMC6074382

[21] Israel E, Hirschwerk D, Jhaveri KD. Acremonium skin and soft tissue infection in a kidney transplant recipient. Transplantation. 2013;95(4):e20. 23423271 10.1097/TP.0b013e31827eefb4

[22] Arievich AM, Minsker OB, Pinzur GS, Fedorchenko TS. Our experience in the treatment of a female patient with generalized ulcerative cephalosporosis of the skin. Vestn Dermatol Venerol. 1968;42(12):63‐65. 5754026

[23] Kan S, Tsai T, Hu C, Lee W. Cutaneous hyalohyphomycosis caused by acremonium in an immunocompetent patient. Br J Dermatol. 2004;150(4):789‐790. 15099392 10.1111/j.0007-0963.2004.05896.x

[24] Texier L, Lahourcade DR, Gauthier Y, et al. Fungal granuloma due to a cephalosporium. Bull Soc Fr Dermatol Syphiligr. 1972;79(5):504‐507. 4676876

[25] Tuon FF, Pozzi C, Penteado‐Filho SR, Benvenutti R, Contieri FL. Recurrent acremonium infection in a kidney transplant patient treated with voriconazole: a case report. Rev Soc Bras Med Trop. 2010;43(4):467‐468. 20802954 10.1590/s0037-86822010000400028

[26] Lee MW, Kim JC, Choi JS, Kim KH, Greer DL. Mycetoma caused by acremonium falciforme: successful treatment with itraconazole. J Am Acad Dermatol. 1995;32(5):897‐900. 7722053 10.1016/0190-9622(95)91557-5

[27] Grunwald M, Cagnano M, Mosovich M, Halevy S. Cutaneous infection due to acremonium. J Eur Acad Dermatol Venereol. 1998;10(1):58‐61. 9552759

[28] Khan Z, Al‐Obaid K, Ahmad S, Abdel Ghani A, Joseph L, Chandy R. Acremonium kiliense: reappraisal of its clinical significance. J Clin Microbiol. 2011;49(6):2342‐2347. 21450966 10.1128/JCM.02278-10 PMC3122737

[29] Tortorano AM, Richardson M, Roilides E, et al. ESCMID and ECMM joint guidelines on diagnosis and management of hyalohyphomycosis: fusarium spp., Scedosporium spp. and others. Clin Microbiol Infect. 2014;3:27‐46. 10.1111/1469-0691.12465 24548001

[30] Guarro J, Gams W, Pujol I, Gené J. *Acremonium**species*: new emerging fungal opportunists–in vitro antifungal susceptibilities and review. Clin Infect Dis. 1997;25(5):1222‐1229. 9402385 10.1086/516098

[31] Krishna V, Morjaria J, Jalandari R, Omar F, Kaul S. Autoptic identification of disseminated mucormycosis in a young male presenting with cerebrovascular event, multi‐organ dysfunction and COVID‐19 infection. IDCases. 2021;25:e01172. 34075329 10.1016/j.idcr.2021.e01172 PMC8161734

[32] Riche CVW, Cassol R, Pasqualotto AC. Is the frequency of candidemia increasing in COVID‐19 patients receiving corticosteroids? J Fungi (Basel). 2020;6(4):286. 10.3390/jof6040286 PMC7712895 33203016

[33] Fekkar A, Poignon C, Blaize M, Lampros A. Fungal infection during COVID‐19: does aspergillus mean secondary invasive aspergillosis? Am J Respir Crit Care Med. 2020;202(6):902‐903. 32687389 10.1164/rccm.202005-1945LE PMC7491399

[34] Messina FA, Marin E, Caceres DH, et al. Coronavirus disease 2019 (COVID‐19) in a patient with disseminated histoplasmosis and HIV—a case report from Argentina and literature review. J Fungi. 2020;6:275. 10.3390/jof6040275 PMC7711963 33182836

[35] Passerini M, Terzi R, Piscaglia M, Passerini S, Piconi S. Disseminated cryptococcosis in a patient with metastatic prostate cancer who died in the coronavirus disease 2019 (COVID‐19) outbreak. Cureus. 2020;12(5):e8254. 10.7759/cureus.8254 32596073 PMC7309194

